# Weighted gene co-expression network analysis of the salt-responsive transcriptomes reveals novel hub genes in green halophytic microalgae *Dunaliella salina*

**DOI:** 10.1038/s41598-020-80945-3

**Published:** 2021-01-15

**Authors:** Bahman Panahi, Mohammad Amin Hejazi

**Affiliations:** 1 Department of Genomics, Branch for Northwest & West region, Agricultural Biotechnology Research Institute of Iran (ABRII), Agricultural Research, Education and Extension Organization (AREEO), Tabriz, 5156915-598 Iran; 2 Department of Food Biotechnology, Branch for Northwest & West region, Agricultural Biotechnology Research Institute of Iran (ABRII), Agricultural Research, Education and Extension Organization (AREEO), Tabriz, 5156915-598 Iran

**Keywords:** Systems biology, Regulatory networks, Computational biology and bioinformatics, Gene regulatory networks

## Abstract

Despite responses to salinity stress in *Dunaliella salina*, a unicellular halotolerant green alga, being subject to extensive study, but the underlying molecular mechanism remains unknown. Here, Empirical Bayes method was applied to identify the common differentially expressed genes (DEGs) between hypersaline and normal conditions. Then, using weighted gene co-expression network analysis (WGCNA), which takes advantage of a graph theoretical approach, highly correlated genes were clustered as a module. Subsequently, connectivity patterns of the identified modules in two conditions were surveyed to define preserved and non-preserved modules by combining the Zsummary and medianRank measures. Finally, common and specific hub genes in non-preserved modules were determined using Eigengene-based module connectivity or module membership (k_ME_) measures and validation was performed by using leave-one-out cross-validation (LOOCV). In this study, the power of beta = 12 (scale-free R2 = 0.8) was selected as the soft-thresholding to ensure a scale-free network, which led to the identification of 15 co-expression modules. Results also indicate that green, blue, brown, and yellow modules are non-preserved in salinity stress conditions. Examples of enriched Kyoto Encyclopedia of Genes and Genomes (KEGG) pathways in non-preserved modules are Sulfur metabolism, Oxidative phosphorylation, Porphyrin and chlorophyll metabolism, Vitamin B6 metabolism. Moreover, the systems biology approach was applied here, proposed some salinity specific hub genes, such as radical-induced cell death1 protein (RCD1), mitogen-activated protein kinase kinase kinase 13 (MAP3K13), long-chain acyl-CoA synthetase (ACSL), acetyl-CoA carboxylase, biotin carboxylase subunit (AccC), and fructose-bisphosphate aldolase (ALDO), for the development of metabolites accumulating strains in *D. salina*.

## Introduction

*Dunaliella salina* is a unicellular halotolerant green alga that can survive in saturated brine (up to 5.5 M NaCl)^1^. This feature makes it an interesting model organism for studying salt tolerance. On the other hand, as the main producer of carotenoids and lipids, *D. salina* is widely used for food and drug industries^2^.

Modifying the environmental circumstances, especially salinity concentration, are among the most effective approach put forth to enhance different metabolites accumulation in these microalgae. However, the biomass productivity of *D. salina* is retarded in a hypersaline conditions^3^, making it difficult to improve metabolite production in large scales.

Exploration of salt stress-responding mechanisms is an inevitable step in resolving these problems. The contribution of ROS and calcium signaling pathway in response to salinity condition has been reported previously^4^. Expressed sequence tag (EST) profiling of *D. salina* in hypersaline condition also identified 1401 unique responsive transcripts were contributed in protein synthesis, energy, primary metabolism, and protein fate^5^. Moreover, a recent global transcriptome sequencing showed that enhancements of photosynthesis and biosynthesis of porphyrins, as well as degradation of starch, synthesis of glycerol, membrane lipid desaturation, are taken part in *D.salina* responses to hypersaline conditions^6^. Hovewer, due to the complexity of salt stresses responding processes in microalgae, underlying molecular mechanisms for salt stress response in microalgae remain a daunting challenge^7^.

There is a lack of integrative investigation on transcriptomes data of *D.salina* under various salinity stress pressures over ranges of treatment periods. Moreover, all of the previous researches have solely focused on differentially expressed genes identification, wherease connectivity analysis has not yet been considered. In contrast to focusing on differentially expressed genes, co-expression module-based network analysis provides new insight into the role of different genes associated with a specific condition, which cannot be detected by standard transcriptome and network analysis^4^. Meanwhile, in these networks, hubs can represent essential genes that may indeed contribute to a specific phenotype^8^. This powerful approach has been widely used on a range of systems mainly in plant species, including Barley^9^, Wheat^10^, Rice^11^, elucidating the comprehensive picture of stress responses. More recently, the efficiency of this approach in identifying groups of expressed genes and highly connected hubs which contributed in secondary metabolites accumulation in microalgae *Auxenochlorella protothecoides* has been confirmed^12^.

The current study focused on co-expression network construction using weighted gene co-expression network analysis (WGCNA) in combination with the identification of hub genes in respective co-expressed modules of salt responsive genes in *D. salina*.

## Materials and methods

### Eligible RNA seq data collection

A search for RNA seq datasets was performed on NCBI Sequence Read Archive (SRA) database using the following keywords: microalgae, Dunaliella, salina, salt, saline, osmotic, stress. Finally, four independent studies that surveyed global transcriptome profiling in *D. salina* at salt stress conditions were selected and raw data were retrieved in fastq format. The first dataset (SRP134914) contains twelve biological samples that were grown in sterile ATCC-1174 DA medium supplemented with NaCl, sorbitol, and H_2_O_2_. We only included the control and NaCl treated samples of this dataset. This dataset was generated by deep sequencing, in triplicate, using Illumina NextSeq500 platform. The second dataset (SRP149387) contains nine samples were generated in triplicate, using Illumina HiSeq 4000 platform. The third dataset (SRP184449) contains twelve samples which were generated using Illumina HiSeq 2000 platform (Table 1).

**Table 1:** Details of datasets and treatment conditions with hypersalinity which were used in this study.

Data set ID	Salinity condition	Sampling time points
SRP134914	NaCl (2 M)	24 h after treatment
SRP149387	NaCl (2.5 M)	6, 12, and 24 h after treatment
SRP184449	NaCl (2.5 M)	0.5, 1 and 2 h after treatment

### Pre-processing and differential expression analysis

Quality control of raw data sets was performed with FastQC (v 0.11.5) software^13^. Adaptor sequence and low-quality reads with Phred score < 30 were trimmed by using Trimmomatic (v0.32) software^14^. The processed reads were subjected to de novo assembly using Trinity (v2.4.0) software^15^ using default parameters. Protein orthology was determined using Blastx (cutoff value of 6) against *C. reinhardtii* and *D. salina* proteins (https://phytozome.jgi.doe.gov/) as described by Dums et al.^16^. The best hits were extracted with in-house python scripts (Supplementary File S1). Filtered reads were aligned to the de novo assembled transcripts using align_and_estimate_abundance Perl script implemented in RSEM (v1.3.1) software^17^. Gene counts were then subjected to Bioconductor DESeq2 package version 1.10.1^18^ to identify differentially expressed genes (DEGs). Comparisons were done using Wald’s test to determine the log2-fold change. To overcome the inconsistency of results of different studies and stabilize the genes’ expression ratios, batch effect correction by using the Empirical Bayes method was performed^19^. This correction enables direct comparisons of expression profiles between biological groups from independent experiments^20–22^. Moreover, genes with low CV less than 10% were filtered out. Finally, common DEGs between four datasets with a threshold |> 1.0| and adjusted p-value < 0.05 were selected to further analysis.

### Weighted gene co-expression network analysis (WGCNA)

Co-expression networks were constructed using the WGCNA algorithm implemented in R WGCNA package^23^. To import selected DEGs in WGCNA package, raw expression values of selected DEGs were normalized with variance StabilizingTransformation (vst) function in R software. Then, a similarity co-expression matrix was calculated with Pearson's correlation $\mathrm{Cor }\left(\mathrm{i},\mathrm{j}\right)$ for all common DEGs. The similarity matrix was transformed into an adjacency matrix (AM) by using the following equation

$$a_{ij} = \left( {0.5{*}\left( {1 + {\text{cor}}\left( {{\text{i}},{\text{j}}} \right)} \right)} \right)^{\beta }$$

where ${a}_{ij}$ denotes the adjacencies between DEGs as a connection strengths index.

The soft-thresholding power beta of the co-expression network was chosen by the criterion of scale-free topology with R2 cutoff (0.8). Finally, the adjacency was transformed into a topological overlap matrix (TOM) and corresponding dissimilarity matrix $(1-TOM)$ using the following formula

$${TOM}_{i,j}=\frac{{\sum }_{u}{a}_{iu} {a}_{uj}+{a}_{ij}}{\mathrm{min}\left({k}_{i},{k}_{j}\right)+1-{a}_{ij}}, {K}_{i}={\sum }_{u}{a}_{iu}$$

where, row index u $(u=1,\dots ,m)$ represents sample measurements.

To obtain co-expressed modules, the parameters were adjusted to minModuleSize = 20 and minimum height = 0.2 to cut the tree.

### Network preservation analysis

ModulePreservation function implemented in WGCNA Bioconductor R package was applied to survey preservation levels of control network modules in the salinity coexpressed modules based on the combination of two preservation statistics including medianRank and Zsummary. Zsummary combines multiple statistics into a single overall measure of preservation that considers density and connectivity aspects of preservation using the following formula

$${Z}_{summary}=\frac{{Z}_{density }+{Z}_{connectivity}}{2}$$

The higher value of a Zsummary indicates the strong preservation in control and treatment conditions. However, the dependency of Zsummary to module size is a challenge, especially when modules with different sizes must be compared. medianRank as a module size independent index is another statistic to test the preservation level. The lower value of a medianRank indicates the strong preservation in control and treatment conditions^23^. Statistical significance of both indexes was tested using permutation testing (here we applied 200 permutations). As prescribed in original reports Zsummary and medianRank were combined and Zsummary < 5 or medianRank < 8 were considered as criteria for considering a module as a non-preserved module^8,23^.

### Identification and validation of hub genes

Hub genes in each co-expressed module were defined according to Eigengene-based module connectivity or module membership (*k*_ME_) index in non-preserved modules. To determine the *k*_ME_, the correlation of expression value of a gene and eigengene of the module were estimated. This index measures the closeness of a gene in a given module. Genes with |*k*_ME_| ≥ 0.7 were considered as hub genes in the respective module^23^. Validation of hubs was performed by using leave-one-out cross-validation (LOOCV) as prescribed in^12^.

### Statistical analysis and functional enrichment

Kyoto encyclopedia of genes and genome (KEGG) Pathway enrichment was performed using Algal Functional Annotation Tool (available http://pathways.mcdb.ucla.edu/algal/index.html)^24^ by setting P-value < 0.05 as a cut-off criterion with *Dunaliella salina* and *Chlamydomonas reinhardtii* genomes as references.

## Results

### DEGs screening

In total, 24 samples of RNA sequencing data were used for identifying DEGs. A total of 4171 common DEGs between control vs. high salinity conditions in three datasets was found (Supplementary file S2) of which 2401 and 1680 were up-and down-regulated in salt stress condition, respectively.

### Identification of modules involved in response to salinity stress

**Figure 1:**
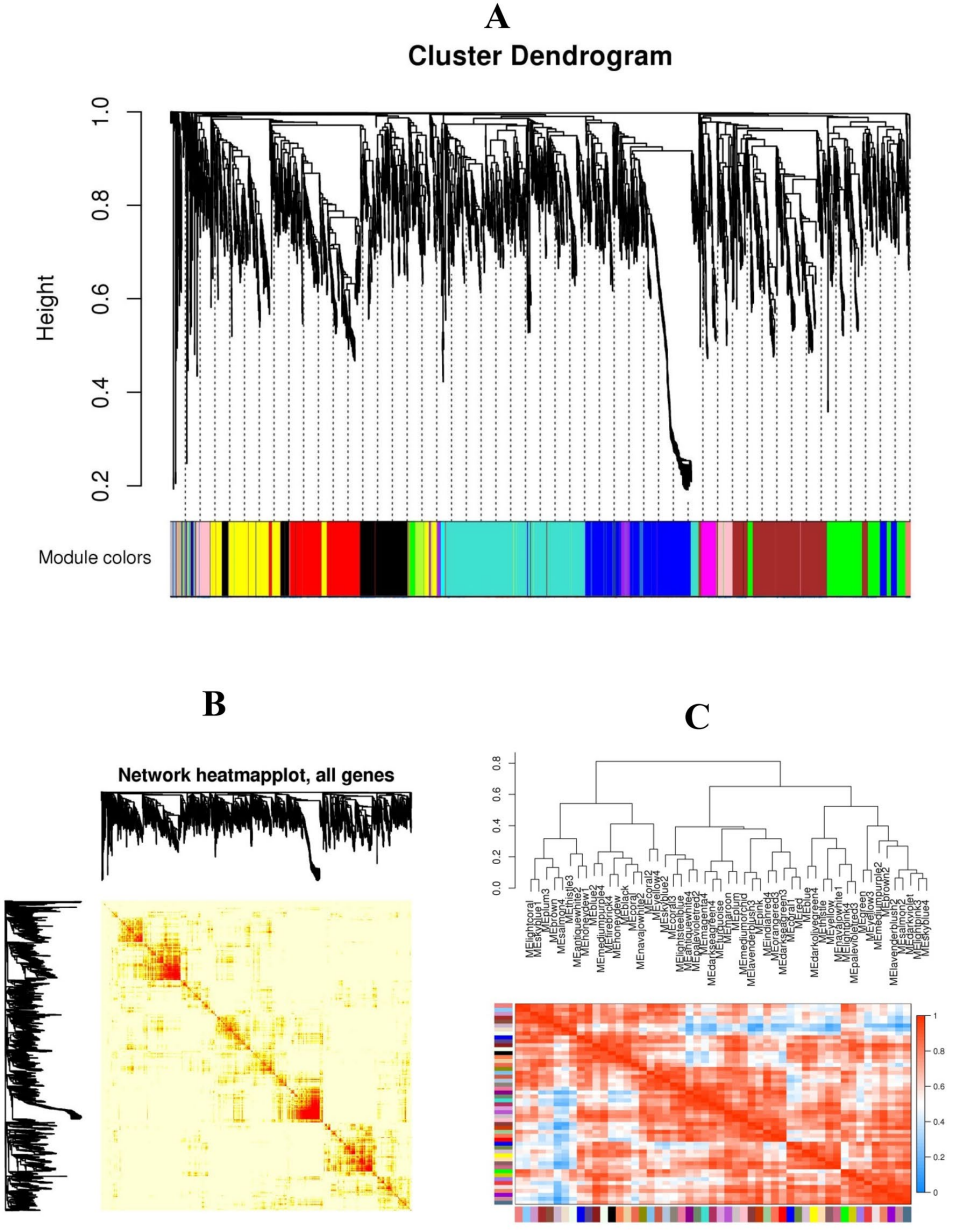
Weighted gene co-expression network analysis of salinity stress responses genes in *D. salina*. (**A**) Hierarchical cluster tree of the common genes between different studies. The branches and color bands represent the assigned module. The tips of the branches represent genes. (**B**) Heat map shows the Topological Overlap Matrix (TOM) value among the proteins of the network delimited in modules by the dynamic method**.** Low TOM is indicated by yellow color and higher TOM is indicated by progressively red color. (**C**) The module Eigen gene adjacency showed by hierarchical clustering and heat map. A module Eigen gene summarizes the gene expression profile of each module. Figures created by the WGCNA Bioconductor package.

By applying the steps described in the “Materials and methods”, two different networks were generated using the genes expressed in control and salt-treated samples, and then, DEGs with similar expression patterns were grouped into modules via the average linkage hierarchical clustering. The adjacency matrix was substituted with the weighted adjacency matrix by raising the correlations to the power of 12, which was chosen using the scale-free topology criterion^23^. We determined the power for which scale-free topology fitting index (R2) was ≥ 0.8 by plotting the R2 against soft thresholds (power β). A total of 15 co-expression modules were identified, which were displayed by different colors (Fig. 1A). The number of genes per module ranged from 21 (cyan) to 872 (turquoise) genes with an average size of 297 genes (Fig. 1A). All genes of each module are represented in Supplementary file S3. Moreover, heat map shows the Topological Overlap Matrix (TOM) value among the proteins of the network delimited in modules by the dynamic method (Fig. 1B). Low TOM is indicated by yellow color and higher TOM is indicated by progressively red color. A module Eigen gene summarizes the gene expression profile of each module was provided in Fig. 1C.

### Network preservation analysis

**Figure 2:**
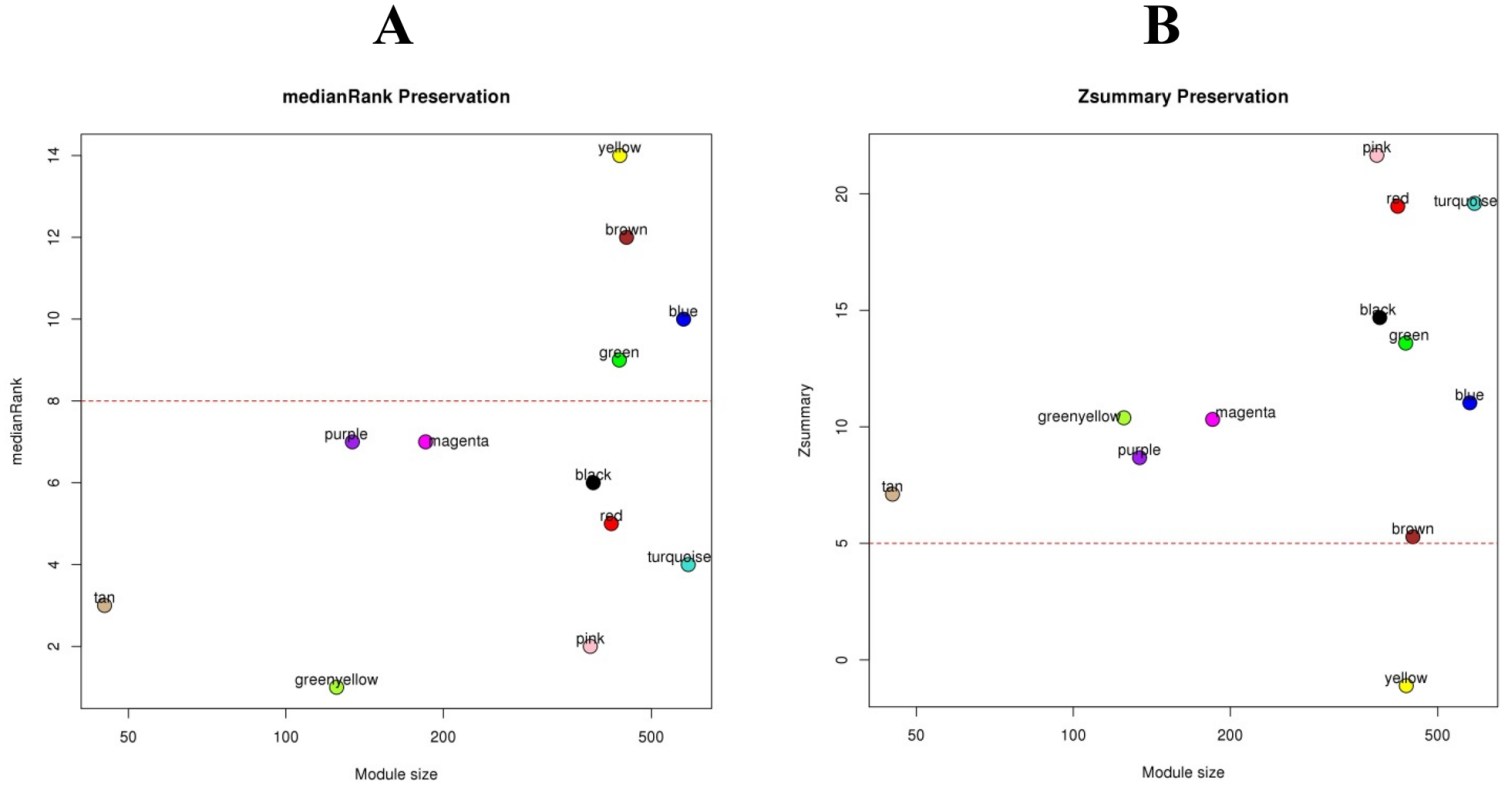
Conservation analysis of defined modules by using medianRank (**A**) and Zsummary (**B**) as a function of the module size. Each labeled color represents a module. The dashed red line indicates the threshold medianRank = 10 and Zsummary = 5. A module was considered as non-preserved if it had medianRank > 8 or Zsummary < 5. Figures created by the WGCNA Bioconductor package.

Preservation analysis of constructed weighted co-expression networks was performed to dissect the connectivity patterns between two control and salinity stress conditions. Connectivity patterns of non-preserved modules are altered under stress conditions compared with control conditions. Preservation statistics of identified modules are shown in Fig. 2. Results showed that eight modules, including purple, magenta, black, red, turquoise, pink, greenyellow, and tan are preserved in control and stress conditions with Zsummary > 5 and medianRank < 8. Whereas, green, blue, brown, and yellow modules were non-preserved.

### Non-preserved modules enrichment analysis

To explore the potential molecular mechanisms responsible for salt stress, we focused on non-preserved modules including green, blue, brown, and yellow modules. Expression patterns and network features of the genes involved in non-preserved modules vary across control and stress conditions. Pathway enrichment analysis was performed using Algal Functional Annotation Tool^24^ and results are shown in Table 2.

**Table 2:** Resulting KEGG pathways enriched in non-preserved modules.

Module	KEGG pathways	Genes
Green	Sulfur metabolism	BPNT1(Dusal.0755s00002.1) ,cysK (Dusal.0059s00009.1), cysl-3 (Dusal.0095s00025.1)
	Oxidative phosphorylation	NDUFV2 (Dusal.1163s00005.1), SDHB (Dusal.0152s00008.1), UQCRFS1 (Dusal.0243s00001.1 )
	Citrate cycle (TCA cycle)	CS (Dusal.0308s00010.1), PDHA (Dusal.0264s00007.1), MDH2 (Dusal.0239s00002.1), SDHB (Cre17.g696600.t1.3)
	Biosynthesis of alkaloids	PDHA, ispH (Dusal.0570s00002.1), CS, dxs (Dusal.0031s00031.1), MDH2 (Dusal.1113s00003.1), SDHB, ilvD (Dusal.0005s00029.1)
	Valine, leucine and isoleucine biosynthesis	VARS (Dusal.0933s00002.1), ilvD, PDHA
	Biosynthesis of phenylpropanoids	CS, PDHA, FBP (Dusal.0670s00008.1), tktA (Dusal.0318s00003.1), MDH2, SDHB
	Glyoxylate and dicarboxylate metabolism	CS, MDH2, MTHFD (Dusal.0454s00008.1)
	Biosynthesis of terpenoids and steroids	Dxs, PDHA, CS, ispH, MDH2, SDHB
	Biosynthesis of plant hormones	ispH, CS, dxs, PDHA, MDH2, SDHB
	Proteasome	PSMC4 (Dusal.0179s00019.1), PSMD6 (Dusal.0579s00008.1), PSMB6, POMP (Dusal.0061s00023.1)
Blue	Ribosome	RP-L11 (Dusal.0453s00002.1**)**, RP-L13 (Cre12.g532550.t1.3), RP-L15 (Dusal.0088s00017.1), RP-L9 (Dusal.0391s00002.1), RP-S5 (Dusal.0942s00006.1), RP-L19 (Dusal.1340s00002.1), RP-L20 (Dusal.0438s00010.1)
	Glycine, serine and threonine metabolism	SHMT (Dusal.0324s00011.1), trpB (Dusal.5270s00001.1.p)
	Selenoamino acid metabolism	ahcY (Dusal.0640s00008.1), MARS (Dusal.0010s00008.1), cysK
	Citrate cycle (TCA cycle)	IDH3 (Dusal.0383s00011.1), ACO (Dusal.0090s00020.1), LSC1 (Dusal.0302s00023.1)
	Aminoacyl-tRNA biosynthesis	MARS, CARS (Dusal.0821s00005.1), IARS, aspS (Dusal.0067s00021.1), GARS
	Vitamin B6 metabolism	thrC, pdxH (Dusal.1073s00003.1)
Brown	Carbon fixation	ALDO (Dusal.0587s00004.1), SBPase (Dusal.0670s00008.1), PRK, GPT, MDH2, rpiA, rbcS (Dusal.1014s00004.1)
	Glyoxylate and dicarboxylate metabolism	MDH2, mdh, rbcS, gyaR (Dusal.0057s00013.1)
	Fatty acid biosynthesis	accC (Dusal.0200s00015.1), KAS2 (Dusal.0826s00002.1)
	Pentose phosphate pathway	ALDO, rpiA (Dusal.1071s00003.1)
Yellow	Porphyrin and chlorophyll metabolism	hemB (Dusal.0026s00019.1), CAO (Dusal.0513s00015.1), CPOX (Dusal.0025s00007.1), HMOX1(Dusal.0838s00005.1)
	Biosynthesis of plant hormones	TM7SF2 (Dusal.0015s00019.1), aspC (Dusal.0001s00030.1), CYP51 (Dusal.0271s00020.1), MDH1, PDHB
	Biosynthesis of terpenoids and steroids	pfkA, CYP51, TM7SF2, MDH1, PDHB

“Biosynthesis of terpenoids and steroids” and “Glyoxylate and dicarboxylate metabolism” were identified as functionally enriched KEGG pathways in both Green and Yellow modules. However, genes that are identified to be part of these pathways are different for Green and Yellow modules. As shown in Table 2, among the genes involved in the biosynthesis of terpenoids and steroids, Dxs (1-deoxy-d-xylulose-5-phosphate synthase), PDHA (Pyruvate dehydrogenase E1 component subunit alpha), CS (Citrate synthase), ispH (4-hydroxy-3-methylbut-2-enyl diphosphate reductase), MDH2 (Malate dehydrogenase), SDHB (Succinate dehydrogenase [ubiquinone] iron-sulfur subunit) genes grouped in Green module whereas pfkA (Pyrophosphate-fructose 6-phosphate 1-phosphotransferase), CYP51 (Sterol 14 desaturase), TM7SF2 (Delta(14)-sterol reductase), MDH1 (Malate dehydrogenase), PDHB (Pyruvate dehydrogenase E1) grouped in Yellow module. Genes involve in glyoxylate and dicarboxylate metabolisms are grouped in two distinct co-expressed modules. Another significantly enriched pathway is “Vitamin B6 metabolism” that is enriched in Blue module and involves thrC (Threonine synthase) and pdxH (Pyridoxine/pyridoxamine 5′-phosphate oxidase) genes. Examples of other enriched pathways in non-preserved modules are “Sulfur metabolism”, “Oxidative phosphorylation”, “Porphyrin and chlorophyll metabolism”, “Carbon fixation”, “Galactose metabolism”, “Carotenoid biosynthesis” and “Fatty acid biosynthesis” (Table 2).

### Hub genes identification and validation

**Figure 3:**
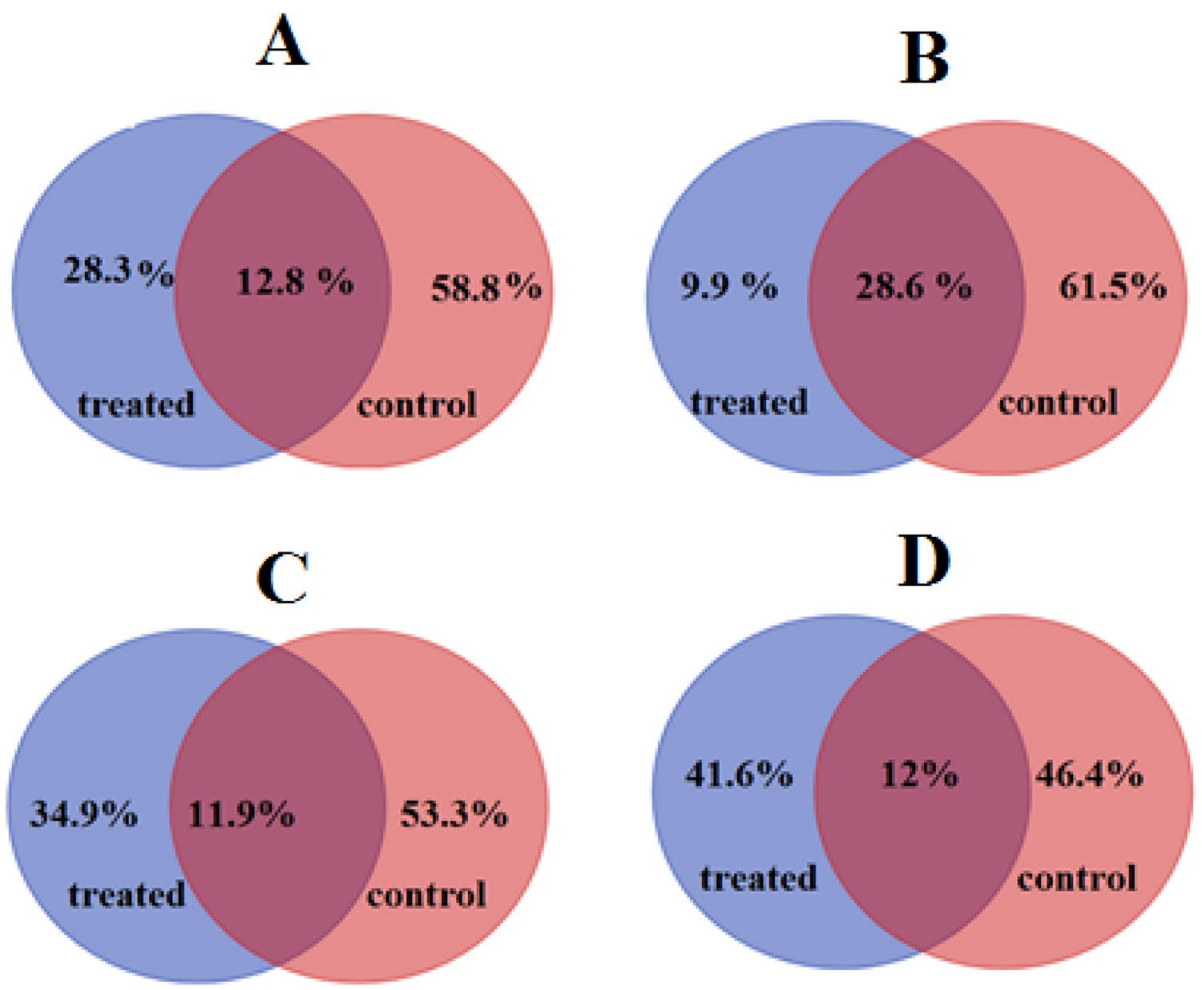
Comparison of hub genes in control and hypersalinity conditions in Green (**A**), Blue (**B**), Brown (**C**), and Yellow (**D**) module as non-preserved modules. Details of identified hub genes for each module can be found in Supplementary File S1.

Hub genes are representative of the module’s overall function and have a high likelihood to be critical components within the module^8,9^. As mentioned above, genes with |*k*_ME_| ≥ 0.7 were considered as hubs in each respective module. Results indicated that the highest and least number of hub genes were in blue and yellow modules, respectively. Comparison of hub genes in control and treated samples of respective modules also showed that brown and yellow modules contained the highest percentages of specific hubs (Fig. 3).

In the green module, 472 genes showed |*k*_ME_| ≥ 0.7 in control condition while these genes had |*k*_ME_| < 0.7 in salinity stress condition (Supplementary file S4). Therefore, these genes were determined as specific hub genes of control condition indicate that the salinity stress disturbed the centrality of these genes. Moreover, 28.3% of identified hub genes in green module were defined as specific salinity specific hubs (Fig. 3A). In the blue module, 9.9% and 61.5% of hubs were defined as salinity and control specific hubs, respectively (Fig. 3B). Moreover, in the brown and yellow modules, 34.9% and 41.4% of identified hubs were salinity specific, respectively (Fig. 3C,D). Using *k*_ME_ value to rank genes in each module, we identified KAS (3-ketoacyl-ACP-synthase) and SHMT (Serine hydroxymethyltransferase 3) in normal condition, as the genes with the highest *|k*_*ME*_*|* value (0.99) However, KAS and SHMT show a structure with low connection with *|k*_*ME*_*|* value = 0.7. These differences highlight the transcendental role of these genes in information transfer in the blue module. To validate hub genes efficiency in distinguishing salinity and control status, the LOOCV method was applied to the expression value of hubs. Results showed that identified hub genes have distinguished two conditions with 94.23% accuracy, highlighting the discriminative power of identified hub genes and validating the defined hubs.

## Discussion

The results of this study showed that topological characteristics of coexpression modules are changed in the salinity network compared to the corresponding modules in the co-expression network of controls. Four non-preserved co-expressed modules were selected for further discussion as mentioned in the result section. Pathway enrichment analysis of these modules can be found in Table 2. It is apparent that several pathways and processes with connection to different mechanisms are contributed to salt stress responses of *D. salina*. Previously transcriptome analysis, which solely has been focused on DEGs identification showed that “post-translational modifications”, “signal transduction mechanisms”; “translation, ribosomal structure, and biogenesis”; and “RNA processing and modification” similarly enriched in both types of analyses^6,25^. Although, systems biology approach applied here rediscovered these processes as responsive mechanisms; however, our analysis showed that some processes such as “sulfur-based metabolisms” and “Proteasome” distinctively enriched in non-preserved modules. Strikingly, our analysis showed that the genes involved in some biological processes such as sulfur-based metabolisms are not only differentially regulated, but also their topology and connectivity are changed in systems levels by hypersaline condition. Previous studies have identified several responsive genes involved in the salt stress condition in *D. salina*. Our study not only confirms these genes contribution in hypersaline condition response, but also WGCNA analysis predicted novel hub genes (Table 3).

**Table 3:** Several previously identified genes which were predicted in non-preserved modules; most of the genes built hub in the modules.

Gene name	Encoded proteins	Hubness	Module	References
BPNT1	Bisphosphate 3′-nucleotidase	Hub	Green	Novel
RCD1	Radical-induced cell death1 protein	Hub	Yellow	Novel
PSMC4	Proteasome 26S subunit, ATPase 4	Non-Hub	Green	Novel
PSMB6	proteasome 20S subunit beta 6	Non-Hub	Green	Novel
IDH3	Isocitrate dehydrogenase	Non-Hub	Green	Novel
PSBP2	Oxygen-evolving enhancer protein 2	Hub	Green	^6,26^
SHMT	Serine hydroxymethyltransferase 3	Hub	Blue	^27^
ALDO	Fructose-bisphosphate aldolase	Hub	Brown	^6^

One of the enriched pathways was sulfur metabolism. Genes responsible for sulfur-based metabolisms, including sulfate assimilation and cysteine- related genes including BPNT1 (bisphosphate 3′-nucleotidase), cysK (Cysteine synthase A), cysC (adenylylsulphate kinase), and cysH (phosphoadenylylsulfate reductase) are co-expressed in *D.salina* in response to salinity stress (Table 1). BPNT1, a member of a structurally related family of phosphatases, is a key regulator of metabolic flow in the sulfur assimilation pathway^28^. Sulfur assimilation is the main process to form sulfur-containing amino acids and metabolites. The up-regulation of BPNT1 might improve the availability of sulfur needed for fast-acting antioxidant systems^29^. Coordinate up-regulation of cysteine- related genes including cysK, cysC, and cysH with BPNT1 highlighted this hypothesis.

Results also showed that salt stress induced protein metabolism. It is apparent that pathways participated in translation (Aminoacyl-tRNA and amino acids biosynthesis) and ribosomal structure significantly enriched in the blue module and proteasome system, as a selective protein and mRNA degradation system, is significantly enriched in Green module. Expression analysis indicated that most of the genes involved in ribosomal structural protein and Aminoacyl-tRNA biosynthesis were up-regulated; suggesting that *D. salina* copes with salt stress by accelerating protein synthesis. On the other hand, the genes encoding proteasome system including PSMC4 (proteasome 26S subunit, ATPase 4), PSMC1(proteasome 26S subunit, ATPase 1), PSMD6 (proteasome 26S subunit, non-ATPase 6), PSMB6 (proteasome 20S subunit beta 6), and POMP (proteasome maturation protein) were up-regulated by salt stress to return the expression of specific genes to normal levels^30^. These findings indicate that a balance between protein synthesis and degradation is one of the main regulatory mechanism coordinating a uniform cellular response to salt stress.

Our results implied that the expression and connectivity of the hub genes within the non-preserved modules were changed by the salinity stress. Therefore, these genes are representative of critical information about the salt stress responding mechanism in *D. salina*. One of the interesting genes that show a large difference of expression and connectivity between salinity and control conditions in *D. salina* is RCD1, which encodes radical-induced cell death1 protein. RCD1 protein is a key regulator of several ROS and salt stress-related transcription factors such as AP2/ERF, NAC, and bHLH^31,32^. Additionally, it has been confirmed that this protein coordinates chloroplast and mitochondrial functions through interaction with ANAC transcription factors in salinity stress^33^. Connectivity analysis showed that |kME| in the salinity stress was higher than 0.8 whereas this value in the control condition was 0.3, indicated that topology and expression of RCD1 were rearranged by hyper salinity condition (Supplementary file S4) to specify protein–protein interactions.

An example of other signaling related gene identified as a salinity specific hub in the brown module is MAP3K13, which encodes mitogen-activated protein kinase kinase kinase 13. Recently, genes coding for four MAPK Kinases (MAPKK) as well as for 10 MAPK kinase kinases (MAPKKK) has been discovered in *D. salina*^34,35^*.* The transcendental role of MAPK proteins in the transduction of stress signaling, cell proliferation, and differentiation through kinase cascades has been confirmed^36^. Moreover, MAPK gene was reported to be involved in *Dunaliella salina* response to the hyper-osmotic and hypertonic shock, suggesting the osmosis regulatory role of MAPK proteins in these microalgae^36^. Cross talk between MAPK signaling with regulation of auxin, ABA, and glycerol biosynthesis pathway has also been highlighted the regulatory impacts of this protein in hyper-saline condition^37^.

**Figure 4:**
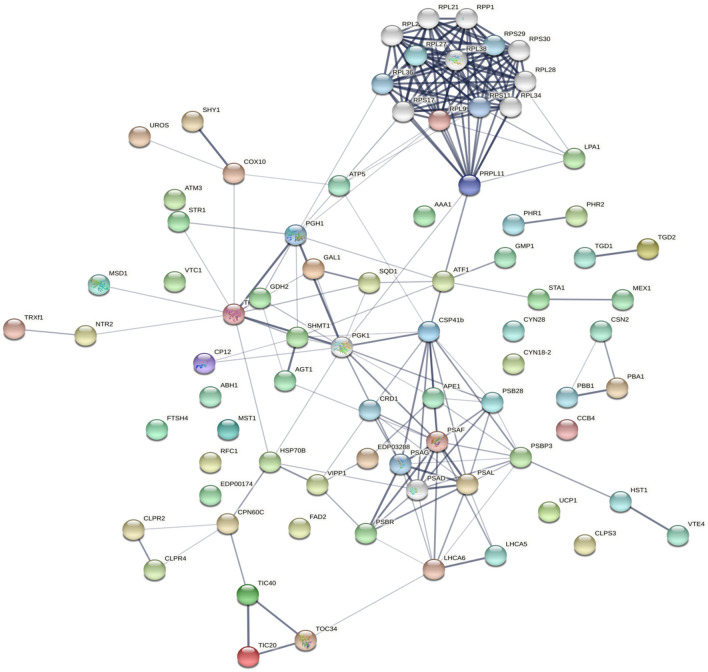
The protein–protein interaction (PPI) network in *D. salina* constructed using STRING database. The abbreviations of protein names are referred to Supplementary Table S5.

**Figure 5:**
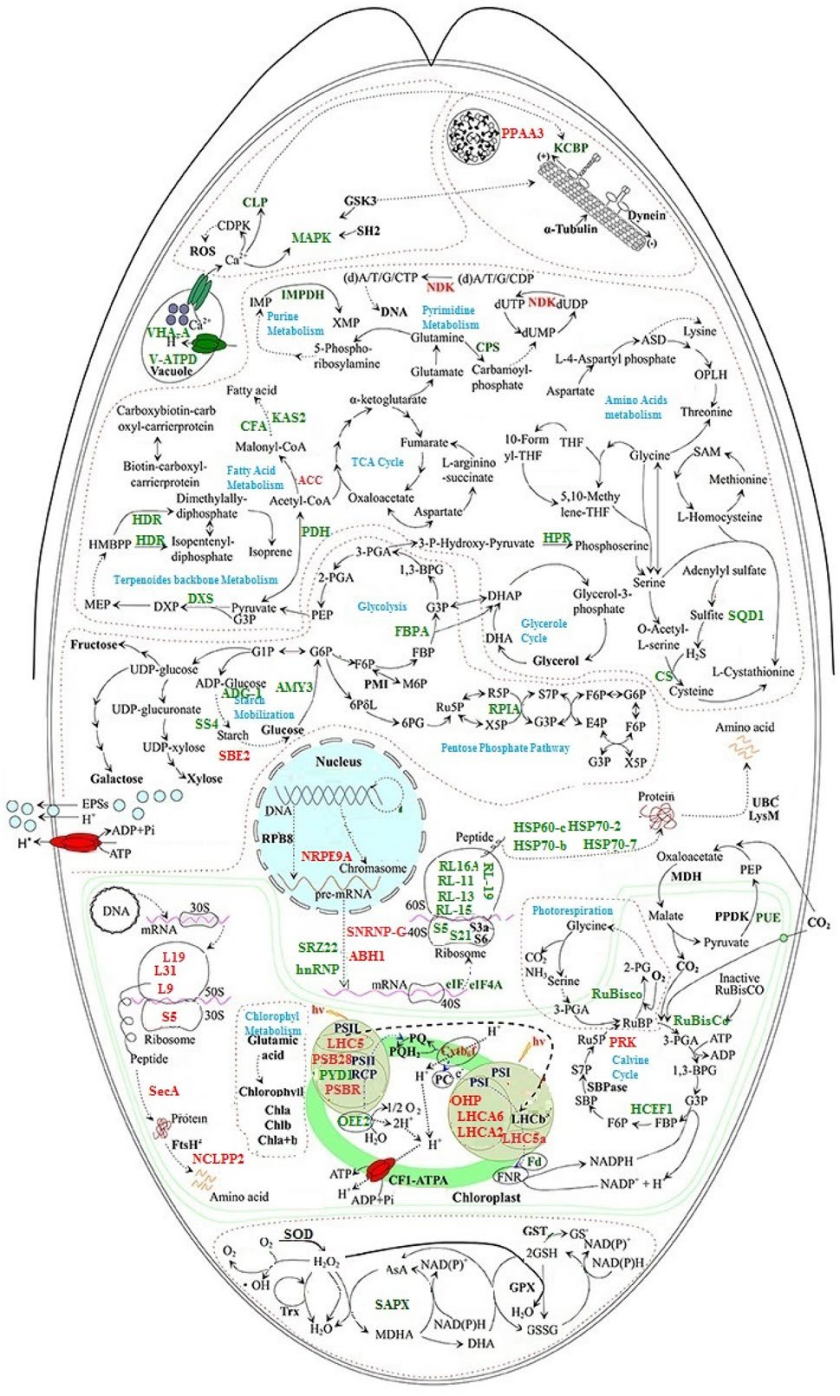
Schematic presentation of the *D. salina* response to salinity stress based on identified hub genes. The solid line indicates single-step reaction, and the dashed line indicates multistep reaction. Hub genes with up regulation and down regulation in response to hypersalinity stress condition written by green and red color, respectively. The abbreviations of protein names are referred to Supplementary Table S5. Figure created by online biorender software available in https://app.biorender.com/.

Another interesting salinity specific hub gene is HSPA1s encoding heat shock 70 kDa protein which is salinity specific hub gene in the blue module. This gene takes part in splicing of newly synthesized mRNAs, folding of de novo synthesized polypeptides, and the translocation of precursor proteins^38–40^. In accordance with our findings, it has been reported that transcript and protein levels of HSP70s in *D. salina* are increased by hypersalinity condition^6,41^ (Figs. 4 and 5). This implies that the transportation and processing of certain newly synthesized peptides into chloroplasts are enhanced by salinity conditions^30^.

ACSL encodes long-chain acyl-CoA synthetase which takes part in PPAR (Peroxisome proliferator-activated receptors signaling) and fatty acid (FA) biosynthesis pathways is another example of salinity specific hub. PPARs are members of the nuclear hormone receptors superfamily, which play a critical role in the expression of many genes regulating cellular differentiation, the metabolism of glucose, and lipids^42^.

PDH (pyruvate dehydrogenase) which takes part in core carbon metabolism is another important hub gene with salinity specific connectivity pattern (Table 2). Recent reconstructed metabolic network for core carbon metabolism revealed the lack of a gene coding for the pyruvate ferredoxin/flavodoxin oxidoreductase and pyruvate formate lyase in *D. salina*^34^. Both enzymes participate in pyruvate metabolism and provide alternative reactions for cells to “bypass” PDH. Lack of the availability of alternatives for pyruvate to acetyl-CoA conversion potentially makes the PDH the bottleneck for carbon flux into fatty acids and lipids in *D. salina*^34^. Specific connectivity pattern shown in current study proposed another potent regulatory layer of PDH to drive carbon into the isoprenoid biosynthesis pathway under salinity condition^43^.

AccC (acetyl-CoA carboxylase, biotin carboxylase subunit) and KAS2 (3-oxoacyl-[acyl-carrier-protein] synthase II) were another salinity specific hub genes that play a critical role in the biosynthetic pathways of nearly all fatty acid (FA)-derived molecules^44^. The genes expression was up-regulated in response to salinity stress conditions which was in agreement with previous finding^4^. Our finding also showed that the change of connectivity pattern of key genes in the fatty acid biosynthesis pathway is another important regulatory mechanism in *D. salina.*

PSBP2 which encodes photosystem II oxygen-evolving enhancer protein 2, which is involved in the regulation of photosystem II, was further considered. Results showed that the expression of this gene was decreased by hypersalinity condition (Fig. 5). Similar findings was also reported by a previous study^6^. Interestingly, this gene was salinity specific hub in the green module, which indicates that not only expression pattern but also connectivity of the mentioned gene was changed by salinity condition.

Fructose-bisphosphate aldolase, ALDO, was another salinity specific hub green module. Fructose bisphosphate aldolase is a key enzyme in photosynthesis; moreover, this enzyme takes part in Glycolysis/Gluconeogenesis, Pentose phosphate pathway, and Biosynthesis of secondary metabolites^45^. It has been shown that a slight change of ALDO activity significantly alters the level of sugars and starch^46^. In agreement with the results of the present study, the up-regulation of ALDO in hypersalinity treated *D.salina* has been reported by previous studies^6,47^, however, topological rearrangement of this gene in salinity stress condition firstly was shown by our WGNCA analysis (Supplementary file S4).

## Conclusion

In summary, our results highlighted the weighted gene co-expression analysis power to shed light on underlying molecular mechanisms by identifying the functional modules and hubs associated with salt stress responses in *D. salina*. Identified non-modules are used for more dissection of contributed pathways in hyper-saline responses, as exemplified by the identified pathways such as “Vitamin B6 metabolism”, “Oxidative phosphorylation”, and “PPARs”. Moreover, the systems biology approach was applied here, proposed some salinity specific hub genes, such as RCD1, MAP3K13, ACSL, AccC, and ALDO (Fig. 5), for development of metabolites accumulating strains in *D. salina*.

## Supplementary Information

Supplementary Information S1.

Supplementary Information S2.

Supplementary Information S3.

Supplementary Information S4.

Supplementary Information S5.
