# Supplementary material for: Weighted gene co-expression network analysis of the salt-responsive transcriptomes reveals novel hub genes in green halophytic microalgae Dunaliella salina
Source: Sci Rep. 2021 Jan 15;11:1607. doi: 10.1038/s41598-020-80945-3 (PMC7810892; doi:10.1038/s41598-020-80945-3)
Supplement: Supplementary file 1 — Supplementary Information S1. [file 41598_2020_80945_MOESM1_ESM.pdf]

Weighted Gene Co-Expression Network Analysis of the Salt-Responsive Transcriptomes Reveals Novel Hub Genes in Green Halophytic Microalgae Dunaliella salina

Bahman Panahi1*, Mohammad Amin Hejazi2

1-Department of Genomics, Branch for Northwest & West region, Agricultural Biotechnology Research Institute of Iran (ABRII), Agricultural Research, Education and Extension Organization (AREEO), Tabriz 5156915-598, Iran

2-Department of Food Biotechnology, Branch for Northwest & West region, Agricultural Biotechnology Research Institute of Iran (ABRII), Agricultural Research, Education and Extension Organization (AREEO), Tabriz 5156915-598, Iran

Supplementary File S1: Python program to extract the geneID from the blastX result

```
#!/usr/bin/python

# Usage get_geneID.py <blast output file>

# Python program to extract the geneID from the blastX result


import sys




def get_geneid():

    inFile = open(sys.argv[1], 'r')

    outFile = open("geneID.txt", 'w')


    outer_query = False

    inner_query = False

    first_newline = False

    tophit = False

    dt_name = ''

    for line in inFile.readlines():

        if outer_query == True and inner_query == True:
```

```
            if first_newline == True:
                if tophit == True:
                    outer_query = False
                    inner_query = False
                    first_newline = False
                    tophit = False
                    continue
                else:
                    geneid = line.split()[0].split('|')[3]
                    outFile.write(dt_name + '\t' + geneid + '\n')
                    tophit = True
            elif line == '\n' and first_newline == False:
                first_newline = True

        elif outer_query == True and 'Sequences producing significant alignments' in line:
            inner_query = True
        elif 'Query=' in line:
            dt_name = line.strip().split()[-1]
            outer_query = True

    inFile.close()
    outFile.close()

if __name__=='__main__':
    if len(sys.argv) < 2:
```

```
    print 'Invalid arguments!\n'
    sys.exit(0)


get_geneid()
print 'I am DONE. Please check the output. :)'
```
